# Increased Frequency of Micronuclei in Adults with a History of Childhood Sexual Abuse: A Discordant Monozygotic Twin Study

**DOI:** 10.1371/journal.pone.0055337

**Published:** 2013-01-30

**Authors:** Timothy P. York, Jenni Brumelle, Jane Juusola, Kenneth S. Kendler, Lindon J. Eaves, Ananda B. Amstadter, Steven H. Aggen, Kimberly H. Jones, Andrea Ferreira-Gonzalez, Colleen Jackson-Cook

**Affiliations:** 1 Department of Human and Molecular Genetics, Virginia Commonwealth University, Richmond, Virginia, United States of America; 2 Virginia Institute for Psychiatric and Behavioral Genetics, Virginia Commonwealth University, Richmond, Virginia, United States of America; 3 Department of Pathology, Virginia Commonwealth University, Richmond, Virginia, United States of America; 4 Department of Psychiatry, Virginia Commonwealth University, Richmond, Virginia, United States of America; 5 Neodiagnostix, Inc, Rockville, Maryland, United States of America; University of Science and Technology of China, China

## Abstract

**Background:**

Childhood sexual abuse (CSA) is a traumatic life event associated with an increased lifetime risk for psychopathology/morbidity. The long-term biological consequences of CSA-elicited stress on chromosomal stability in adults are unknown. The primary aim of this study was to determine if the rate of acquired chromosomal changes, measured using the cytokinesis-block micronucleus assay on stimulated peripheral blood lymphocytes, differs in adult female monozygotic twins discordant for CSA.

**Methods:**

Monozygotic twin pairs discordant for CSA were identified from a larger population-based sample of female adult twins for whom the experience of CSA was assessed by self-report (51 individuals including a reference sample). Micronuclei (MN) contain chromatin from structurally normal or abnormal chromosomes that are excluded from the daughter nuclei during cell division and serve as a biomarker to assess acquired chromosomal instability.

**Results:**

Female twins exposed to CSA exhibited a 1.63-fold average increase in their frequency of MN compared to their nonexposed genetically identical cotwins (Paired *t*-test, *t*_16_ = 2.65, *P* = 0.017). No additional effects of familial factors were detected after controlling for the effect of CSA exposure. A significant interaction between CSA history and age was observed, suggesting that the biological effects of CSA on MN formation may be cumulative.

**Conclusions:**

These data support a direct link between CSA exposure and MN formation measured in adults that is not attributable to genetic or environmental factors shared by siblings. Further research is warranted to understand the biological basis for the observed increase in acquired chromosomal findings in people exposed to CSA and to determine if acquired somatic chromosomal abnormalities/somatic clonal mosaicism might mediate the adult pathology associated with CSA.

## Introduction

Childhood sexual abuse (CSA) not only compromises well-being in childhood but is also associated with a broad range of psychopathology and morbidity in adulthood [1], [2], [3], [4]. However, little is known about the biological mechanisms involved in mediating the long-term pathogenic effect of early-life trauma. One possible means for CSA to be biologically “embedded” in a manner that could lead to a latent pathologic consequence would be if it resulted in a change in the individual’s somatic cell DNA. Evidence shows childhood maltreatment predicts an increased risk of clinically relevant levels of inflammation in adulthood [5],[6], and inflammation-associated reactive oxygen/nitrogen species are known to cause DNA damage/chromosomal changes. Stress-related inflammation also leads to perturbations in the regulation/expression of several genes, including (but not limited to) nuclear factor-kappa B (NF-kβ), interleukin-1B, interleukin-6, and tumor necrosis factor-α [7], [8]. Additional evidence that early-life stress can lead to DNA-based alterations comes from reports of shortened telomeres in children exposed to adverse rearing settings [9], and in adults with a history of chronic or severe childhood illness [10] or childhood maltreatment [11], but the potential correlation between childhood maltreatment and telomere length is controversial [12].

Given that both telomere shortening and/or inflammation-related generation of oxygen and/or nitrogen species are phenomena that have been associated with an increased frequency of acquired chromosomal abnormalities [13], [14], [15], [16], [17], [18], [19], we hypothesize that an alternative or additional biological effect of stress could be the acquisition of somatic cell chromosomal instability. Moreover, since such damage can cause mutations and chromosomal abnormalities that disrupt cellular function and viability through aberrant gene expression and protein formation [20], [21], [22], the accumulation of chromosomal imbalances over time provides a plausible account by which CSA exposure could contribute to the development of later health and psychiatric symptoms observed in adults with a CSA history.

**Figure 1:**
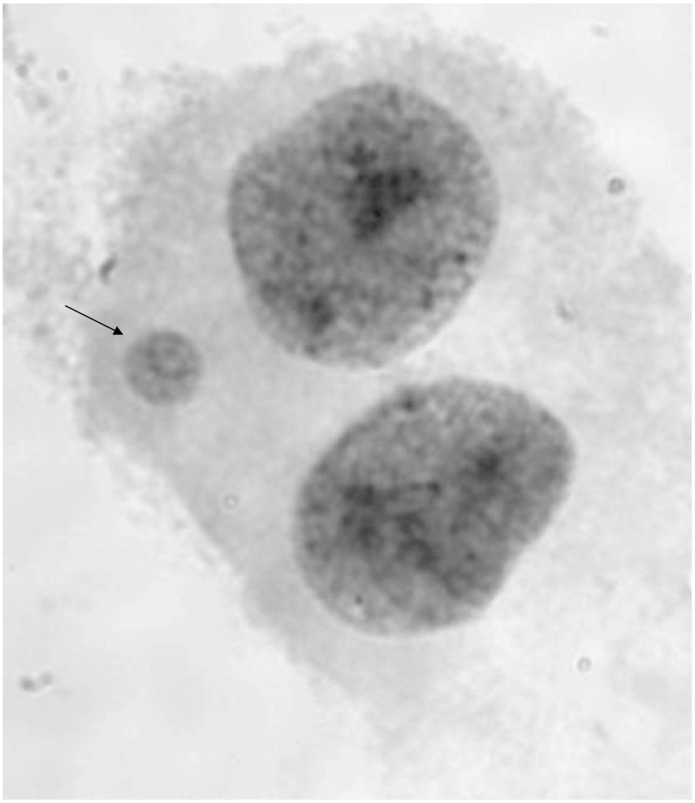
Giemsa stained micronucleus (MN) (arrow) and corresponding daughter binucleates. By definition, a MN is no larger than one-third the size of the parental nuclei and appears adjacent to the binucleate.

The cytokinesis-block micronucleus (CBMN) assay is an attractive biomarker for estimating chromosomal damage associated with environmental insults or exposures and is considered an acceptable alternative to data obtained from the assessment of metaphase chromosomal analyses [23], [24], since it is less labor intensive and less prone to producing artifacts than classical chromosomal studies [25]. Briefly, a micronucleus (MN) is a small chromatin-containing structure that can be visualized juxtaposed to the main daughter nuclei following the completion of mitosis (Figure 1). MN formation can occur spontaneously or in response to environmental exposures and can accumulate over several months or years [26]. MN form when whole chromosomes or chromosomal fragments fail to correctly migrate to spindle poles during mitosis [27], [28], [29]. The lagging chromosome(s) or fragment(s) are subsequently excluded from the daughter nuclei and are encased in their own nuclear envelope [27]. The exclusion of chromatin into a MN can result in alterations of cellular gene dosage, which, in turn, could result in abnormal gene expression and/or perturbations in cellular proliferation that could have a broad cascade of consequences on biological systems [30]. MN frequency is known to increase with age [23], [31], [32] and has been shown to be elevated in patients with several health conditions, including cancer [33], cardiovascular disease [34], [35], Alzheimer’s disease and Parkinson’s disease [36].

MN frequency is influenced by both heritable genetic and environmental factors [32], [37]. However, the extent to which MN frequency is impacted by exposure to a traumatic event, such as CSA, is not known. One of the most robust approaches to determine the causal role of non-genetic influences on trait variation is to study monozygotic (MZ) twins who are discordant for exposure histories. Theoretically, because the DNA of MZ twins differs only for induced changes, they provide a unique opportunity to study the long-term biological impact of childhood traumatic events.

In the present study we tested the hypothesis that adult females who experienced CSA have a higher frequency of spontaneously occurring leukocyte MN than their identical twins who did not experience CSA. Although these twin pairs are quite rare, we elected to use a discordant identical twin study design since it allows one to control for known genetic influences on MN formation [32], [37] and provides an effective means for separating the causal effects of CSA from background familial risk factors known to associate with CSA.

## Methods

### Ethics Statement

Human subjects research was approved by the Virginia Commonwealth University IRB (#12407 and #179). Written informed consent was obtained from all research participants.

### Sample and Assessment of Childhood Sexual Abuse

Female-female CSA discordant MZ twins were ascertained from the population based Virginia Adult Twin Study of Psychiatric and Substance Use Disorders [38] and the Mid-Atlantic Twin Registry (MATR) at Virginia Commonwealth University (VCU), with the details of ascertainment outlined elsewhere [39], [40], [41]. Briefly, the pairs derive from two related samples, born from 1934–1974, who were eligible to participate in the previous study [4] if both members responded to a mailed questionnaire (response rate was about 64%). Eighty-eight percent of the participants in the original study were first interviewed face to face in 1987–1989 (at which time their mean [SD] age was 30.1 [7.6] years [range, 17.0–55.0 years]). They were subsequently interviewed three additional times by telephone with at least a one year interval between follow-ups. The remaining 12% were first interviewed face-to-face in 1992–1994 and assessed a second time (with the same interview given to the rest of the sample during the fourth wave) by telephone in 1996–1997. During the second-wave interview, twins were queried about their willingness to respond to questions about CSA and their preferred method of assessment. Most preferred a mailed self-report questionnaire, which was employed using items developed by Martin et al. [42]. The initial item was:

“Before you were 16, did any adult, or any other person older than yourself, involve you in any unwanted incidents like (i) inviting or requesting you to do something sexual, (ii) kissing or hugging you in a sexual way, (iii) touching or fondling your private parts, (iv) showing their sex organs to you, (v) making you touch them in a sexual way or (vi) attempting or having sexual intercourse?”

Of the 1411 individuals completing this portion of the interview, there were 326 MZ twin pairs where both twins provided information about CSA exposure, of which 74 pairs were classified as discordant for CSA. For the present study, specimens were collected from 17 of these discordant MZ twin pairs (Table 1) in which one twin endorsed none of the CSA items and the other twin fell into one of three exclusive, hierarchical exposure categories: (1) *non-genital* (*N* = 3 pairs) [numbers (i), (ii) and (iv)], (2) *genital* (*N* = 8 pairs) [numbers (iii) and (v)] and (3) *intercourse* (*N* = 6 pairs) [number (vi)]. The mean age at the time of the first CSA incident was 10.7 years-old (s.d. = 3.9) and ranged from 5 to 16 years of age. These CSA discordant MZ twin pairs were invited to complete a health history questionnaire and submit blood samples (VCU IRB #12407). After providing their informed consent, blood samples were obtained by a health care provider of the participants’ choosing and shipped to our cytogenetics laboratory (overnight delivery carrier) at room temperature per standard procedures. A random sample of age-matched female MZ twins (7 pairs plus 3 individuals without a cotwin [*N* = 17]) was also obtained from the MATR to serve as an unselected reference sample (VCU IRB #179), with health history questionnaire completion and blood specimen processing occurring using the identical protocol. The mean current age of the discordant MZ twin pairs (Mean = 48.8, SD = 9.7) was not significantly different from that of the reference sample (Mean = 53.7, SD = 9.4) (*t*-test, *t*_29_ = 1.47, *P* = 0.154).

**Table 1:** Rates and characteristics of the specific forms of childhood sexual abuse experienced by the affected individuals, their communication/support experience, and their perpetrator’s status (*N* = 17).

Childhood sexual abuse	%	Perpetrator status	%
CSA type		Female	0.06
* Sexual invitation (i)* *	75.0	Multiple individuals	18.8
* Sexual kissing (ii)*	62.5	Forced or threatened you	47.1
* Fondling (iii)*	70.6	Age of perpetrator(s)	
* Exposing (iv)*	60.0	*<15 y*	15.0
* Sexual touching (v)*	26.7	*15–18 y*	35.0
* Intercourse (vi)*	37.5	*19–24 y*	20.0
		*25–49 y*	15.0
After these incidents:		*>50 y*	15.0
* I told no one*	81.3		
* I told someone and* *was believed and supported*	17.6	Relationship with perpetrator	
		* Relative living at home*	17.6
* I told someone and was* *believed but not supported*	0.06	* Non-relative living at home*	0.0
		* Relative not living at home*	6.0
* I told someone and was not* *believed, blame, or punished*	0.0	* Family friend or other important*	29.4
		*adult not living at home*	
* Telling someone put an* *end to the abuse*	100.0	* Acquaintance or neighbor*	41.2
		*Stranger*	17.6

* Type as listed in Methods sub-section, ‘Sample and Assessment of Childhood Sexual Abuse’. 70.6% of affected individuals experienced more than one CSA type. Participants were classified into three exclusive, hierarchical exposure categories: (1) *non-genital* (*N* = 3 pairs) [numbers (i), (ii) and (iv)], (2) *genital* (*N* = 8 pairs) [numbers (iii) and (v)] and (3) *intercourse* (*N* = 6 pairs) [number (vi)].

### Assessment of Adult Psychopathology

A number of psychiatric and substance use disorders were assessed multiple times in the discordant twins using DSM-IIIR [43] or DSM-IV [44] criteria. Lifetime diagnosis of major depression, generalized anxiety disorder and alcohol and other drug dependence was assessed at the fourth interview by trained interviewers [35]. Lifetime panic disorder was assessed at earlier interviews only (waves 1 and 3). Further details of the diagnostic algorithms and diagnostic reliability can be found in Kendler et al. [38].

### DNA Isolation and Zygosity Determination

Twin zygosity status was confirmed, using genomic DNA that was isolated from whole blood using the Puregene DNA Isolation Kit (Qiagen), based on the patterns of 13 highly polymorphic short tandem repeat sequences (AmpFlSTR® Profiler Plus® and Cofiler® kits, Applied Biosystems, Foster City, CA).

### Cell Culture

To ensure that erythrocytes did not confound the recognition and scoring of MN, leukocytes were isolated using Histopaque-1077 (Sigma) and then established in culture according to standard procedures (RPMI 1640 media supplemented with 15% fetal calf serum and the mitogen phytohemagglutinin) [45]. Forty-four hours after initiation of the cultures, cytochalasin-B was added (3 µg/ml final concentration). Cells were harvested at 72 hours using standard techniques, including a 10-minute incubation in hypotonic solution (0.075 M KCl), and serial fixation (three times in 3∶1 methanol: acetic acid solution) [45]. Slides were made following standard procedures [46].

### CBMN Assay

MN were visualized following Giemsa staining (4% Harleco Giemsa solution) and identified according to the criteria established by Fenech et al. [47] (Figure 1). The frequencies of MN observed in the cytochalasin-B blocked binucleated cells of the twins were calculated by averaging the values obtained from two replicate scores (1000 binucleates were evaluated from two independent areas of the slide for a total of 2000 binucleates per study participant). Given that differences in nuclear proliferation could impact observed MN frequencies, the nuclear division cytotoxicity index (NDCI) was calculated using Fenech’s adaptation of the protocol of Eastmond and Tucker [45], [48], as follows: NDCI = [Ap+Nec+M1+2(M2) +3(M3) +4(M4)]/N, where Ap = the number of apoptotic cells; Nec = the number of necrotic cells; M1; M2; M3; and M4 = the number of cells having 1, 2, 3, or 4 nuclei, respectively; and N = total number of cells scored (viable as well as non-viable). The cytogeneticists were blinded to twin pair membership and CSA exposure status.

### Statistical Analysis

Differences in MN rates and NDCI values in the CSA exposed twins versus their nonexposed cotwins were assessed by a paired Student’s *t*-test. A general effect of CSA, not differentiated by severity of exposure, was examined in all tests since this was deemed appropriate based on the current literature [1], [3], [4]. A variance stabilizing square root transformation was applied to the MN frequency data for pairwise analyses, given that it was reasonable to assume the distribution of MN scores follow a Poisson distribution. The Wilcoxon signed rank test was also used as a nonparametric equivalent to the paired *t*-test since data transformations were not required and it provided an additional safeguard against biases sometimes encountered with modest sample sizes. Two-sided *P*-values were reported for all pairwise comparisons and exact *P*-values were calculated for non-parametric tests.

One could speculate that the nonexposed twin of pairs discordant for CSA could have elevated MN levels because of exposure to other shared adverse family factors not directly related to CSA or potentially from stress arising from knowledge that her cotwin was abused. To further test whether an effect of CSA on MN levels was restricted only to the abused twin a population sample of age-matched MZ female twins was incorporated in the analyses to serve as a reference group. Tests were performed using generalized mixed-effect models [49] with Poisson error distribution adjusting for covariance within families and the effect of age. Two fixed effect terms were included to specify the relevant contrasts among the different twin exposure classes. A CSA exposure term was coded positive for CSA exposed twins and negative for CSA nonexposed and reference sample twins. An additional term to indicate exposure to adverse familial environment was created where discordant pairs were coded as positive and reference sample twins as negative. Evidence for a CSA related family adversity effect beyond that of direct CSA exposure would be indicated by a significant coefficient for the second term while controlling for any influence of the first term. These models were also used for additional tests exploring differences in the rate of MN formation by age between CSA exposed and nonexposed twins. All analyses were performed using the R statistical programming language [50].

## Results

### Pair-wise Comparisons

**Figure 2:**
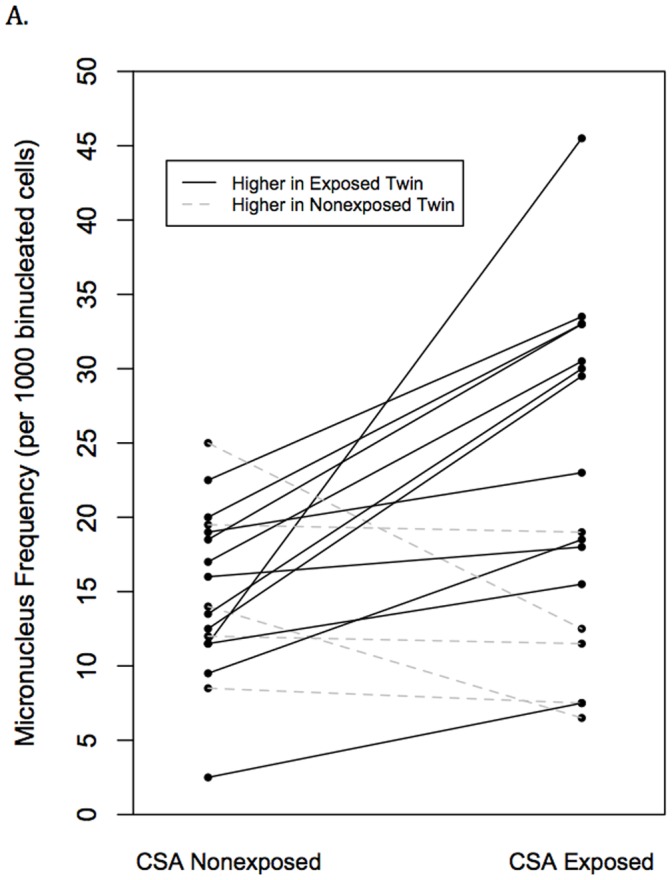
Pairwise comparison of (A) MN frequencies (*t*_16_ = 2.65, *P = *0.017) and (B) nuclear division cytotoxicity index (*t*_16_ = 0.66, *P = *0.518) in CSA discordant MZ twin pairs (*t*_16_ = 2.65, *P = *0.017).

MZ twins exposed to CSA exhibited on average a 1.63-fold increase in the occurrence of MN compared to their nonexposed cotwin. The absolute MN frequency values were greater in the CSA exposed twins for 12 of the 17 discordant twin pairs (Figure 2A). Furthermore, the slope of the comparison line was near zero for 3 of the 5 pairs where their MN level was nominally higher in the CSA nonexposed twins. To determine if there might be differential levels of cellular proliferation/viability between the cotwins, their NDCI values were compared using a paired *t*-test, but showed no significant difference (*t*_16_ = 0.66, *P = *0.518) (Figure 2B). In contrast, a paired *t*-test comparison indicated a significantly higher frequency of MN formation in the CSA exposed twins (*t*_16_ = 2.65, *P = *0.017) and resulted in a significant Wilcoxon rank sum test (*W = *124, *P = *0.023). We then tested if these results were largely influenced by the discordant twin pair having the largest difference in MN level (the steepest slope in Figure 2A) by removal of this pair and repeating the analysis. This repeat assessment also showed a significant increase in MN frequency in the twins exposed to CSA (*t*_15_ = 2.38, *P = *0.031). CSA discordant cotwins did not differ on measured diet and lifestyle factors and no significant differences were found for rates of adult disease (Table 2). It should be clarified that the present sample was not designed to replicate the modest odds ratios reported by Kendler et al. between CSA exposure and the presence of adult psychiatric and substance disorders [4]. The goal of performing these latter tests was to examine whether the presence of adult health/behavioral conditions might confound the association between CSA status and MN formation.

**Table 2:** Lifestyle characteristics and adult psychiatric and substance use disorders in CSA discordant MZ twin pairs.

	CSAExposed^1^	CSA Nonexposed^2^	Both Endorsed	Neither Endorsed	Odds Ratio^3^	*P* *
Medication Use^4^	1	3	7	5	0.3	0.63
Green, Leafy VegetableIntake (5 days per week)	1	6	4	5	0.2	0.13
Smoking Status						
Lifetime (>50 cigarettes)	1	1	5	5	1.0	0.50
Last 30 days (>15 days)	2	0	0	10	+∞	0.50
Heart Disease	1	1	0	13	1.0	1.0
High Blood Pressure	2	2	1	10	1.0	1.0
Cancer Diagnosis	0	2	0	13	-∞	0.50
Alcohol Dependence	2	1	0	14	2.0	1.0
Any Drug Abuse or Dependence	2	0	0	15	+∞	0.50
Lifetime Depression	5	2	5	5	2.5	0.45
Lifetime Generalized Anxiety Disorder	1	0	0	16	+∞	1.0
Panic Disorder	2	0	0	12	+∞	0.50

1 indicates pairs discordant for CSA and item where the exposed twin was positive for the item (n_21_).

2 indicates pairs discordant for CSA and item where the nonexposed twin was positive for the item (n_12_).

3 odds ratio for twin pairs doubly discordant for CSA and item (n_21_/n_12_).

4 prescription and non-prescription use for more than 1 year excluding birth control.

+/− ∞, value is positive/negative and infinite due to a null value in at least one category.

* two-sided P value from exact binomial test.

### Group Comparisons

**Figure 3:**
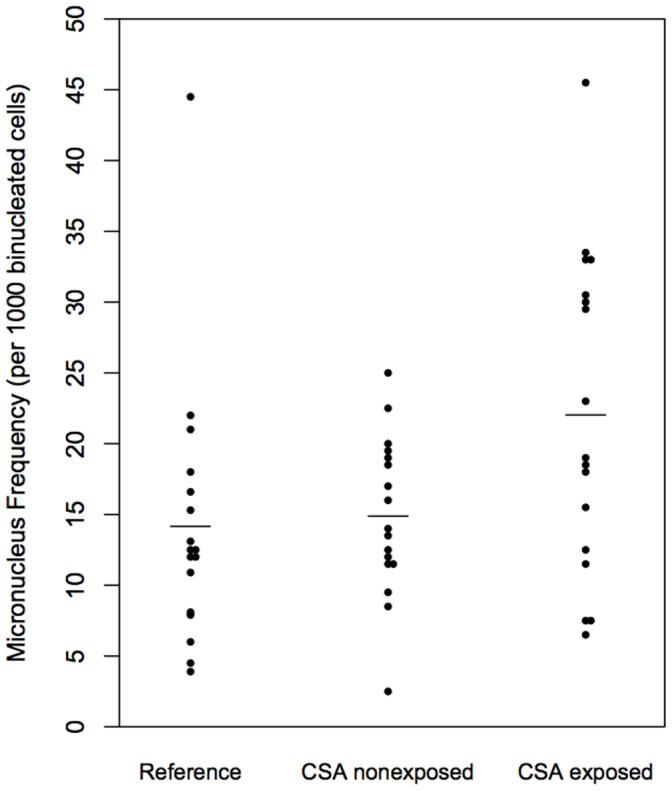
MN frequencies for discordant MZ twin pairs and age-matched controls. The mean level is indicated by a horizontal bar for controls (mean [SD] 14.2 [9.4]), CSA nonexposed (14.9 [5.6]) and CSA exposed (22.0 [11.3]) twins. CSA exposure status was significant in this combined sample (*P*<0.001), while there was no indication of an additional effect attributable to the familial environment (*P = *0.406) based on results from generalized mixed-effect models.

The overall mean [SD] MN frequency in CSA exposed twins was 22.0 [11.3] compared to 14.9 [5.6] per 1000 cells in their nonexposed cotwins (Figure 3). The mean MN level in the unselected reference twins (14.2 [9.4]) was not significantly different from that of the CSA nonexposed set. While CSA exposure status was highly significant in this combined sample (*P*<0.001), there was no indication of an additional effect attributable to the familial environment (*P = *0.406) based on results from generalized mixed-effect models. Removal of the most extreme value in the reference sample (greater than 3 standard deviations from the mean) resulted in a reduction of the mean MN level to12.3 and a more similar estimate of variability as the CSA nonexposed sample (SD = 5.4), but still yielded similar modeling results as the full sample.

**Figure 4:**
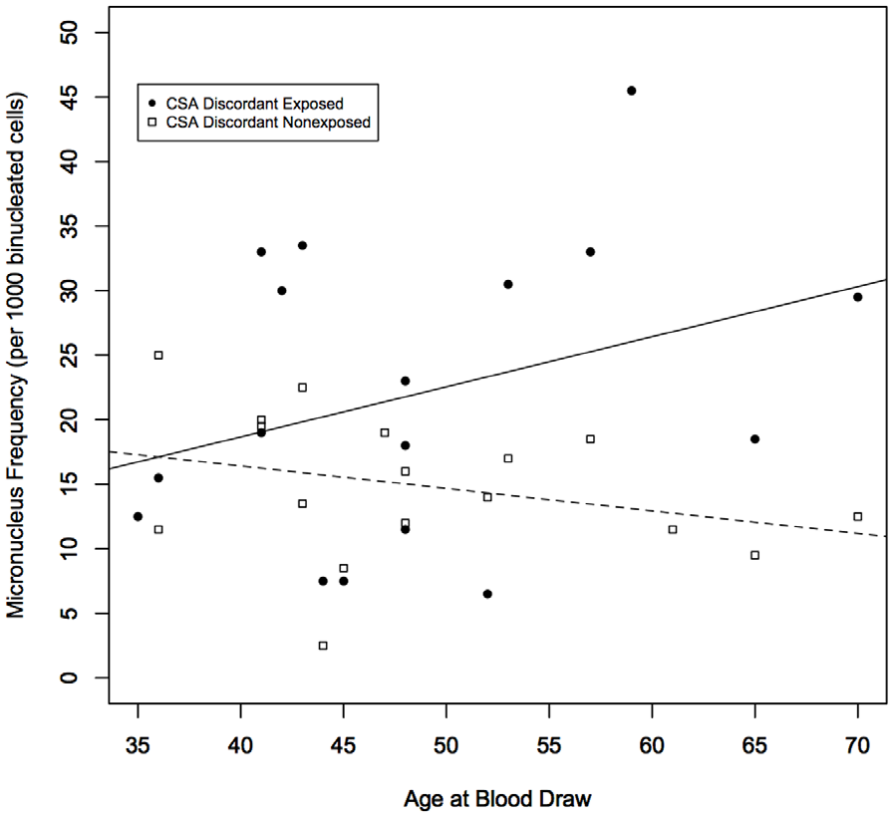
Relationship between MN frequency and age for CSA exposed and nonexposed twins. A significant interaction effect was observed (coefficient [SE] = 0.030 [0.009], *P* = 0.0006) with the MN level in CSA exposed twins increasing with age while the MN level remained constant across the limited age range evaluated in CSA nonexposed twins.

Not surprisingly, the clearly established association of age with MN that has been shown by our lab and several previous investigators was not identified in this study, as estimated using mixed-effect models (*P* = 0.661), since the twins selected for study were from a narrow age range. However, the difference in MN frequencies between discordant cotwins showed a significant interaction with age, with the divergence increasing over time (coefficient [SE] = 0.030 [0.009], *P* = 0.0006) (Figure 4). Separate tests of age on each group indicated the interaction between age and CSA exposure was driven primarily by a significant increase of the level of MN in the CSA exposed group (coefficient [SE] = 0.017 [0.005], *P* = 0.001) rather than a decrease in MN level for the CSA nonexposed group (coefficient [SE] = −0.012 [0.007], *P* = 0.072). There was no increase in MN frequency with age over the limited age-matched range evaluated for the reference group (*P* = 0.361).

## Discussion

The present study yielded three main findings. Firstly, female twins exposed to CSA had an increased frequency of acquired somatic chromosomal changes (measured using MN), compared to their nonexposed, genetically identical (MZ) cotwins. Secondly, our analyses ruled out the hypothesis that the higher MN level was due to an indirect association through other shared familial risk factors. Thirdly, evidence was found for an increase in MN level with age in the CSA exposed twins that was not observed in their genetically identical nonexposed cotwins. This latter finding suggests that there may be a cumulative effect of CSA exposure on the frequency of acquired MN in lymphocytes.

### CSA is Associated with MN Formation

The short- and long-term negative sequelae of CSA have been extensively documented. Kendler, et al. [51], who recently reported that the impact of environmental experiences, including traumatic event exposure, contributes substantially to stable and predictable inter-individual differences in symptoms of depression and anxiety that are observed by middle adult life. Although a number of possibilities exist, the biological mechanisms whereby childhood adversity “gets under the skin” to result in latent adult health/behavior consequences have not been well established [52], [53]. For example, there is ample support for the association of chronic psychological stress with persistent sensitizations of the hypothalamic-pituitary-adrenal axis and autonomic stress response [6], [53], [54]. The atypical development of stress reactivity could bring forth direct changes leading to acquired chromosomal abnormalities in lymphocytes through the induction of inflammatory factors known to influence MN formation, such as IL-6 [55]. Investigators have also shown that an increase in inflammatory activity can elicit the formation of reactive oxygen species, through the deleterious effects of chronically elevated glucocorticoid levels [55]. The resulting oxidative stress may lead to DNA or telomere damage/chromosomal aberrations, resulting in MN formation. Alternatively, epigenetic changes in genes associated with the cascade of biological responses to stress may lead to perturbations in mitotic apparatus formation, chromosomal alignment and/or DNA synthesis, which could subsequently lead to chromosomal malsegregation [55], [56], [57], [58], [59]. Evidence that methylation changes influence acquired chromosomal abnormality frequencies comes from studies of hypomethylated cells obtained following either: (1) *in vitro* induction (primarily using 5-azacytidine); or (2) as a result of mutation (cells from patients having immunonodeficiency, centromeric heterochromatin instability, and facial anomalies [ICF] syndrome, which is a condition in which the individuals have a mutation in the DNA methyltransferase 3b gene). The results of these investigation have shown increases in the rate of micronuclei associated with methylation alterations (particularly chromosomes 1, 9, and 16 in the samples from people having ICF), with observed delays in centromere separation being suggested as at least one means whereby the observed increase in somatic chromosomal abnormalities was acquired [59], [60], [61], [62], [63], [64].

### The Accumulation of Damage Across the Lifespan

Interestingly, we observed a significant statistical interaction whereby only twins exposed to CSA displayed an increased MN level over the limited age range evaluated in this study. This association suggests that these individuals accumulated chromosomal changes over their lifespan, with this increase appearing to be in addition to normal, age-related or stochastic events. It is unlikely that this increase was limited to an effect of the normal aging process [21], [29], since it was not also seen in identical cotwins who were not exposed to CSA; nor was it seen in the negative control reference group. Extrapolating from data collected in our previous study of MN frequencies in healthy individuals [32], the “biological age” of the CSA exposed twins was inferred to be 9.9 years older, on average (95% CI, 2.8–17.1), than their CSA nonexposed cotwins. The accumulation of chromosomal instability acquired over the lifespan provides one plausible explanation for the non-specific adverse health effects of child maltreatment and suggests a framework for general susceptibility to a wide variety of adult illnesses and mental health outcomes. Alternatively, rather than chromosomal instability being causally related to the latent health problems observed in adults experiencing CSA, its presence could serve as an accessible and easily measured proxy for recognizing other biologically relevant changes that have occurred and could place the individual at an increased health risk.

### Methodological Strengths and Limitations

In this study, the relationship between CSA and MN formation was tested using a powerful model; the discordant MZ twin design. Perhaps the most significant advantage of this approach is its ability to address issues related to direction of causality within cross-sectional data. Given that CSA has been shown to correlate with multiple family background risk factors, nearly all of which are shared by twins (such as interpersonal loss, family discord, and economic adversity), the discordant MZ twin design was effective for controlling for the effect of these influences. Without sufficient control in epidemiological samples, which necessitates the measurement of all confounding factors (some of which are unknown) the clustering of childhood adversities would likely serve to overestimate the association between CSA and MN formation. Similarly, the use of MZ discordant twins served as a control for factors related to MN formation that could be shared by twins, including but not limited to, inherited defects in genome maintenance [56], [57]. Another strength of this study design was our sampling of discordant twins who are currently in mid to late adulthood, thereby allowing an appreciable time for the accumulation of stress-related cellular damage to arise from an early life trauma (CSA occurred before 16 years of age). Aspects of this study that are novel include: (1) the conjecture that chromosomal instability could serve as a candidate system for the dysregulation of biological systems that could be “remembered” and accumulated through multiple cell divisions over time and; (2) the use of MN frequency as a potential biomarker for acquired biological changes that have accrued following CSA.

Although our discordant MZ twin study design provided a powerful test of our primary hypothesis, this investigation had methodological limitations. For instance, since we studied lymphocytes, the observation of an increased frequency of acquired chromosomal changes in twins experiencing CSA might have the greatest relevance to health problems associated with the cascade of biological effects mediated through the inflammatory system and may or may not be directly applicable to the acquisition of adult psychopathology. Indeed, this tissue sampling limitation may explain, at least in part, the lack of a clear relationship between MN frequency and the presence of adult psychopathology/morbidity for the limited number of conditions evaluated in our sample. However, it is of interest to note that chromosomal changes, primarily aneuploidy, are acquired in many tissues, normal brain and nerve cells, throughout development and that the brain and other somatic cells from individuals having a variety of health and/or psychiatric conditions show higher levels of acquired chromosomal abnormalities than controls [20], [65], [66], [67].

### Summary

In summary, our study results showed increases in acquired chromosomal instability in female twins exposed to CSA, with the effect appearing to be cumulative with age, and independent of the family environment. Improvements in our understanding of this and other biological changes associated with CSA could lead to the development of biomarker panels for identifying individuals who are most at risk for acquiring health problems. In addition, these persisting biological alterations underscore the gravity of sexual abuse in children.
